# Medical Opinion and Movement

**Published:** 1909-02-06

**Authors:** 


### Medical Opinion and Movement.

THE precipitin reaction for hydatid cysts, discovered by Drs. Welsh and Chapman, has been the subject of a more extended investigation at their hands and those of Mr. J. C. Storey, the results of which are published in the *Australasian Medical Gazette*. Their first communication dealt merely with observations which showed that a special precipitation reaction can be obtained by mixing the serum of a patient suffering from hydatid disease with the fresh fluid from one of his cysts. To make the test of any value in diagnosis it is evident that more is required: the experiments now reviewed were designed to find out whether the reaction takes place when fluid from the cysts of another patient is used; whether fresh fluid is absolutely necessary; and, if not, how best to preserve such fluid in a sterile condition. As a result of experiments on animals, and with the sera of 20 patients unquestionably suffering from hydatid cysts, they conclude that a precipitate is not obtained in any interaction between hydatid fluid and serum from a patient not invaded by hydatid cysts; but that a certain number of negative reactions occur, although such cysts are present. The positive value of the precipitin test is greater than the negative, that is to say, and is of great clinical importance. It is also to be noted that not all hydatid fluids interact equally well with the sera of hydatid patients, and that some appear to fail altogether; and that sterile hydatid fluid if kept for a great length of time slowly loses its capacity for interacting with serum in this way. Further, the authors emphasise the fact that great differences exist between the hydatid antiserum arising in the human subject in conditions of accidental and usually chronic infections, and that prepared by the artificial inoculation of animals in which the subsequent morbid processes are much more acute.

---

THE vaccine treatment of typhoid fever forms the subject of an article contributed to the *Medical Record* by Drs. Watters and Eaton, of Boston, who have employed the method in 30 cases. They used a stock emulsion of bacillus typhosus, made and standardised by themselves, and sterilised by moist heat at 60° C., and by 0.3 per cent. lysol. In no instance did any unfavourable result occur, clinically or pathologically, even when no definite improvement could be demonstrated. The doses given varied between 15 and 60 millions, and were oftenest 25; sometimes but one dose was exhibited, sometimes as many as four or five, and always by injection into the subcutaneous tissue overlying the biceps muscle The authors illustrate the effects of their treatment by reproducing the whole 30 temperature charts, and they remark that clinically the symptoms were generally found to ameliorate even before the defervescence of the fever took place. Certainly the charts, which represent 30 consecutive and totally unselected cases, seem to show a very marked effect of the vaccine injection in most of the patients. A negative phase of short duration is often discernible, followed rapidly by a positive phase, during which the temperature reaches normal by lysis. In some cases this sequence was not apparent, or not fully apparent, after a single dose, so one or more repetitions in increased quantity were then given. Two out of the 30 patients died, but one of them is stated to have been comatose on admission, and virtually a hopeless case from the first. Hæmorrhage occurred but once; perforation not at all. The authors recognise, in a truly scientific spirit, that their experience is too small for any generalisations to be based upon it; they publish the series of cases for what they are worth, and to encourage others to make trial of the method so that the truth about its merits or demerits may the sooner be arrived at.

---

A CASE of bronchial calculus is described by Dr. Farr in *International Clinics*, and serves as a text for an interesting summary of this very rare affection. These calculi may originate in the lung tissue itself or in cavities from calcification of tubercular, pneumonic, or actinomycotic deposits; but more often they arise in the bronchi, where foreign bodies, plugs of fibrin, or blood-clot may serve as nuclei for their production. West believes that Sir Robert Carswell was right in assigning calcification of a bronchial gland with subsequent ulceration into the lung or bronchi as the commonest method of origin. Other observers have demonstrated bone and cartilage in bronchial calculi, and have inferred thence that calcified bronchial cartilages may be a cause of them. The condition may be entirely latent, and the calculus may be expelled during an attack of bronchitis or pneumonia. More often there are chronic symptoms whose duration may be as long as five years. Cough is frequently paroxysmal, but dyspnœa differs from that of asthma by being mainly inspiratory. Pain is common, and may radiate from the chest to the arm of the affected side. Hæmorrhage is also frequent and often recurrent, but it is rarely large in amount. Bronchitis and pneumonia are very common complications. The prognosis is said to be good in non-tuberculous cases. The patient whose case is described was at once relieved of symptoms which had lasted eighteen months when she expectorated her calculus, and has remained well for two years since. No calculus has yet been diagnosed before expulsion, notwithstanding the use of *x*-rays, which failed to reveal the stone in this case. Expectorants are said to be entirely valueless in treatment.

---

DR. H. VOGEL draws the attention of the profession to the fact that a unilateral affection of the lung is frequently accompanied by elevations of temperature localised to the affected side. This fact has already been pointed out by Peter and others, but appears to have attracted little consideration on the part of clinicians. After carrying out a large number of observations on patients affected with a pulmonary lesion, Dr. Vogel is led to the conclusion that this difference in temperature between the two axillæ occurs with few exceptions, and may amount to 0.5° C. In cases of bilateral pulmonary lesions the temperature is generally higher on the side where the changes are more accentuated and are developing more rapidly. In cases in which a lesion of one lung of some long standing is followed by an affection of the other lung, which has hitherto remained intact, the axillary temperature will be higher on the side of the more recent affection, and in general, where both lungs are affected, the differences of temperature are indications of the relative activity of the disease on the two sides. Injections of tuberculin are said to cause a disappearance of these differences of temperature, but on increasing the dose the relation between the temperatures of the two sides may actually be reversed, so that the temperature of the affected side is lower than that of the healthy.

---

The field for original research in tropical medicine and parasitology is still immense, and it seems a most deplorable thing that jealousy and recrimination should arise between individual workers. Not long ago it was Sir David Bruce and Dr. Castellani who could not see eye to eye about certain events in Uganda, and now it is Professor Looss, of Cairo, and Dr. Sambon whose controversy has degenerated into acrimony over questions connected with the Schistosomes. The latter maintains that the ova of the true Schistosomum hæmatobium are always terminal spined; what have been described as the imperfect or lateral spined ova of this worm he holds to be a distinct species, which he calls Schistosomum mansoni. He describes these ova as both longer and broader than those of the S. hæmatobium, and of more oval shape compared with the lanceolate form of the latter. The lateral spined parasites are never found in the bladder, but mainly in the large intestine, being voided in the fæces in large quantities. This species alone, he believes, is endemic in the West Indies and South America, and also in parts of the Congo Free State, while in Cape Colony only the terminal spined variety is known. Elsewhere in Africa both kinds exist and may occasionally be demonstrated in the same individual. Into the details of the squabble between the two helminthologists over these points it is unprofitable to follow, for the tone in which the dispute is being argued out is one which all true scientists must deplore.

---

Heredity in diabetes mellitus is the subject of an article by Dr. R. T. Williamson in the *Medical Chronicle*. The author finds that he can trace a family history of diabetes in 22 out of the last 100 cases which he has seen in private, and he regards this as the minimum percentage which careful inquiry is likely to reveal. Assurance companies which demand information as to a family history of tuberculosis and cancer very seldom trouble about diabetes, though it appears to Dr. Williamson most desirable that they should. A family history is very important, he says, if more than one near relative has suffered, if the proposer is very stout, and if his mode of life is unsatisfactory in respect of alcoholism, worry, sedentary occupation, or excessive indulgence in saccharine foods. In any such person he thinks much might be done to postpone or prevent the onset of diabetes by a *régime* designed to correct any of these factors which may be present. The relatives of diabetic patients in whom the disease is most frequently found are brother, mother, father, sister. In 250 cases of diabetes there were five instances of a husband and wife both exhibiting the disease. The physical and mental strain of nursing a diabetic husband may possibly have been the explanation of the cases in which the wife developed symptoms later than the husband. In other cases it has been thought possible that both have been subject to the same severe mental strain or worry; or both may have taken excess of alcohol or sugar; or both may have lived under unsatisfactory hygienic conditions.

---

In a recent article in the *Deutsche Med. Woch.* Dr. W. Pust advocates the use of plaster of Paris in the treatment of infected wounds, which was suggested to him by a consideration of the destructive action upon micro-organisms of desiccation. He has obtained excellent results with the following procedure. The skin around the wound is first thoroughly cleansed, and a compress of aseptic muslin placed on the ulcer and maintained there by a band. A thick layer of plaster is then applied to this, and covered with aseptic cotton wadding and gummed paper, the whole being kept in place by a few turns of bandage. The layer of plaster is renewed frequently for several days, until the wound has become thoroughly cleansed. The hygroscopic action of the plaster produces a kind of mechanical extraction of the pathogenic organisms from the depths of the tissues. He supposes that disinfection is brought about partly by the desiccation of the organisms and partly by the centripetal current of the discharges, which carries the organisms away from the deeper layers of the wound.

---

There have been many attempts to produce an efficient anti-pneumococcal serum, but until quite recently the activity of such preparations has been of the feeblest. Landmann, of Darmstadt has, however, now introduced a polyvalent serum which seems to have come very well through the official tests of the Ehrlich Institute of Experimental Pathology. The serum, as far as animals are concerned, appears to have prophylactic as well as curative effects: relatively small doses saved an animal as late as eight days after experimental infection with a virulent culture of pneumococci. Moreover the polyvalence of the serum would seem to be established as far as concerns the different species of organism which Landmann was able to procure. The successful results obtained experimentally give rise to hopes of equal success in the treatment of the disease in human beings. It must be remembered, however, that curative sera should not be reserved solely for the treatment of desperate cases. Just as is the case with antidiphtheritic serum the best results are obtained in pneumonia when Landmann's antitoxin is used early in the disease. Landmann invites clinicians to treat a certain number of cases of unilobular pneumonia with his serum and an equal number of similar cases without it with the object of finding out if it tends to check extension of the disease to another lobe. No ill effects have as yet followed the use of this serum. The author recommends that 20 c.c. should be injected daily as a curative measure, and a rather less quantity for a prophylactic dose.

---

PAUL CARNOT and Deflandre have discovered important variations in the character of the blood during and after the menstrual period. From the first day of the period, even when the flow is very scanty, a diminution of red corpuscles can be noticed, which is accentuated on the following days. The diminution reaches its maximum about the fourth or fifth day and often amounts to more than a million per cubic millimetre. After the cessation of menstruation, the number of red cells rises gradually to the normal in from 10 to 12 days. The authors will not venture an opinion as to whether this represents a real loss of red cells, due partly to the hæmorrhage and in part to some form of hæmolysis. They point out that the diminution in red cells may be merely apparent and due to a redistribution of the cells. The poverty of the blood in red corpuscles may be due to the intense congestion and hyperæmia of the genitalia. This diminution of red cells, real or apparent may have some relation to the tendency to syncope and vertigo and the lowness of general resisting power seen in so many women during the catamenial period.

---

CALMETTE and Guerin have recently communicated a paper to the Académie des Sciences of Paris describing a new medium for the cultivation of the bacillus of tubercle. This medium consists of specially prepared bile, made up into a solution containing 5 per cent. bile in glycerine. The technique of the preparation of the medium is too lengthy for description in these columns, and the authors' experiments for the same reason cannot be described here. Their communication, however, is of sufficient interest for their conclusions to be given at some length. These are as follows:—The bovine bacillus of tubercle, cultivated on the bile of cows, acquires special characteristics which are wanting in bacilli cultivated on the ordinary media. Bacilli from these bile-media are easily absorbed through the intestinal wall, and when absorbed in sufficient quantity by this route create lesions which quickly calcify. When injected intravenously into animals of the bovine species these cultures produce a general febrile disturbance without the formation of tubercles. Human bacilli can be cultivated only with difficulty on the bile of cows, and bacilli derived from birds will not develop at all on such a medium. If, however, human and birds' bile be substituted for cow's bile, the respective bacilli will grow easily and will take on the same characteristics as bovine bacilli grown on cow's bile. It would therefore seem that this new method of cultivation can be employed advantageously for the differentiation of tubercle bacilli into species.

PULMONARY bilharziosis is discussed at length in the *Transvaal Medical Journal* by the medical officer to the Witwatersrand Native Labour Association, Mr. G. A. Turner, who has systematically examined the lungs for the ova of the bilharzia parasite in a large series of consecutive postmortems. In 33 natives who died of pulmonary complaints, 17 of which were tuberculosis and 13 pneumonia, 23 were found to harbour the ova in their lungs, and nine of these contained them in great numbers. Of 26 who died from other causes, seven had pulmonary bilharziosis very extensively and seven more on a lesser scale. Out of the whole number of 59 bodies, 52 had bilharziosis of the bladder. All the ova found were of the terminal-spined variety, with the exception of one lateral-spined ovum found in the lung of a man who died of some nodular hepatic condition which is stated to have been either tubercular or syphilitic. Most of the natives in question came to Johannesburg from the East Coast districts of the Transvaal and of Portuguese East Africa, and the remainder from Nyassaland and other tropical regions. Mr. Turner suggests the possibility that the transference of these negroes from the low-lying districts where they are recruited to the lofty plateau of the high veld, 6,000 feet above the sea, may determine both the respiratory diseases which chiefly account for the mortality and the lodgment of the ova in the lungs. It is suggested that owing to this ascent the accumulation of eggs in the pelvis is disturbed, and some of them are turned loose into the blood stream, by which they are then distributed.

---

COBBOLD, it is pointed out, long ago suggested the possibility that bilharziosis is responsible for the peculiar susceptibility of the South African native to pneumonia. It is certainly reasonable to suppose that a lung stuffed with quantities of the gritty calcareous bilharzia ova must be unduly vulnerable to the attacks of the pneumococcus and the tubercle bacillus. Another important bearing of Mr. Turner's researches is upon the question of hæmoptysis, which, bearing in mind the results of bilharzial infection of the bladder, might be expected frequently to follow its extension to the lungs. Scrapings of tubercular cavities have yielded the ova, and therefore presumably they may also be found in the sputum, though it seems the author has not searched there for them. It is worth remarking, too, that the usual process of staining films for tubercle bacilli involves the use of a decolourising solution of sulphuric acid, which dissolves bilharzia ova almost instantaneously; it follows that special search is required for these parasites in separate films. On the question of treatment the author inclines to the use of soamin, which he has lately started to use in all pneumonia cases; but he has not yet had sufficient experience of the results to enunciate any conclusions about its value. He points out, also, that as most of the patients harbour malarial parasites in addition to others the use of preparations containing arsenic is advantageous on that ground alone.

